# More severe joint disease and lower patient oxygenation are associated with less corrosive in vivo synovial fluid in patients with knee osteoarthritis

**DOI:** 10.1302/2046-3758.152.BJR-2025-0019.R1

**Published:** 2026-02-03

**Authors:** Baptiste Ulrich-Ischer, Anna Igual Muñoz, Yueyue Bao, Geneviève Perrenoud, Stefano Mischler, Brigitte M. Jolles

**Affiliations:** 1 Lausanne University Hospital and University of Lausanne (CHUV-UNIL), Department of Musculoskeletal Medicine, Swiss BioMotion Lab, Lausanne, Switzerland; 2 Tribology and Interfacial Chemistry Group, Institut des Matériaux, École Polytechnique Fédérale de Lausanne, Lausanne, Switzerland

**Keywords:** Corrosion, Prosthesis, Synovial fluid, Knee osteoarthritis, Disease severity, total knee arthroplasty (TKA), knee, inflammation, Western Ontario and McMaster Universities Osteoarthritis Index, patient-reported outcome measures, stiffness, BMI, joint disease

## Abstract

**Aims:**

Electrochemical properties of synovial fluid are variable among patients and can lead to implant corrosion, negatively impacting their longevity. The purpose of this study was to explore the relationships between electrochemical properties of synovial fluid of knee osteoarthritic (OA) patients undergoing total knee arthroplasty (TKA) and their clinical and demographic data.

**Methods:**

Knee OA patients undergoing TKA were enrolled in this study, and samples of their synovial fluid were collected during surgery and immediately injected into a three-electrode electrochemical cell to measure their electrochemical properties, including open circuit potential, polarization resistance, and cathodic current density. Synovial fluid samples from 43 patients were collected (25 females; mean age 69.9 years (SD 7.6); mean BMI 27.6 kg/m^2^ (SD 5.2)). Clinical evaluation of the patients was conducted preoperatively to assess the disease severity, the inflammation in the knee joint, and patient-reported outcomes. A correlation analysis was performed to study the relationship between the electrochemical parameters of the synovial fluid and demographic and clinical data of the patients.

**Results:**

Significant correlations were found between disease severity and both the polarization resistance and the cathodic current density, between WOMAC stiffness scores and polarization resistance, and between KSS knee scores and both open circuit potential and cathodic current density. Finally, patients with a history of oxygen-reducing medical conditions had larger open circuit potential than patients without this kind of medical history.

**Conclusion:**

For the first time, correlations between patients’ characteristics clinical and an in vivo electrochemical measurement have been obtained. The results showed that patients with more severe disease and more symptoms had less corrosive synovial fluid. Moreover, this study showed lower corrosive properties of synovial fluid in patients with a history of oxygen-reducing medical conditions, highlighting the critical role of oxygen in corrosion.

Cite this article: *Bone Joint Res* 2026;15(2):113–120.

## Article focus

- Electrochemical properties of synovial fluid are variable among patients and can lead to implant corrosion, negatively impacting their longevity.
- We conducted a correlation analysis between electrochemical properties of in vivo synovial fluid of knee osteoarthritic (OA) patients undergoing total knee arthroplasty and their clinical and demographic data.

## Key messages

- Disease severity was correlated with electrochemical properties of the synovial fluid, indicating that the higher the disease severity, the less corrosive the synovial fluid.
- Correlations were found between electrochemical properties and symptoms, with the synovial fluid being less corrosive in patients who had more symptoms.
- The synovial fluid of patients with a history of oxygen-reducing medical conditions was less corrosive.

## Strengths and limitations

- For the first time, correlations between patients’ clinical characteristics and an in vivo electrochemical measurement have been obtained.
- While the study provides valuable insights into the relationship between electrochemical properties of in vivo synovial fluid and demographic and clinical data of patients with knee OA, further studies are necessary to confirm the results.

## Introduction

Joint arthroplasty is a widely performed surgical procedure, with more than three million knee and hip prostheses implanted in the USA in the last ten years. The number of procedures has increased in recent years and is expected to reach over five million prostheses implanted in 2040.^1^ While these procedures considerably improve a patient’s quality of life, the longevity of implants remains a critical concern, with more than one-third of the prostheses being implanted before 65 years of age.^1^

The introduction of metal into the human body, as occurs with joint arthroplasty implants, inevitably raises concerns regarding its long-term stability in a biological environment. Although metallic implants are engineered for durability, they can undergo corrosion when exposed to body fluids.^2-4^ This corrosion process leads to the release of metal ions (such as cobalt, chromium, and titanium) into surrounding tissues and the systemic circulation.^5-8^ These ions are associated with various adverse effects, including local inflammation, pseudotumour formation, metallosis, and even systemic reactions.^9,10^ Moreover, corrosion compromises the longevity of the implant, and in severe cases, these complications necessitate prosthesis revision surgery, imposing substantial physical and emotional burdens on patients as well as economic costs to healthcare systems.^11^

While most attention regarding implant corrosion has historically focused on hip arthroplasty, total knee arthroplasty (TKA) is not exempt from corrosion-related complications and metal ion release. Multiple studies have demonstrated that knee implants, particularly with modular components or metallic interfaces, are subject to in vivo degradation. Lons et al^5^ conducted a prospective study showing a statistically significant increase in circulating levels of cobalt, chromium, and titanium ions one year after TKA, despite favourable clinical outcomes. Corrosion and wear damage have also been documented in long-term retrieved cobalt-chromium femoral components, with Arnholt et al^2^ identifying several cases of inflammatory cell-induced corrosion. In modular knee prostheses, a study observed taper corrosion at junctional interfaces and reported adverse local tissue reactions, such as osteolysis and pseudotumour-like inflammation, in a subset of patients requiring early revision.^12^ More recently, a study confirmed that titanium-titanium taper junctions in TKA also undergo corrosion, exhibiting damage patterns comparable to those observed in hip prostheses.^13^ In a comprehensive review, the same group highlighted the various biological responses to metal debris in the knee, including genotoxicity, immune activation, and hypersensitivity reactions.^14^ This is consistent with a systematic review that identified metallosis as a rare but serious complication in knee arthroplasty, often related to implant failure or polyethylene wear, and emphasized the importance of early diagnosis and revision when necessary.^11^ Collectively, these findings underscore the need to better understand corrosion mechanisms and metal ion behaviour in the knee, in order to improve implant longevity and minimize biological complications.

The composition of synovial fluid, which bathes the joint and the prosthetic interface, plays a pivotal role in modulating the corrosion risks of implanted materials.^15^ Synovial fluid is a complex biological medium composed of serum proteins, hyaluronic acid, lipids, and lubricine,^16^ all of which can influence the electrochemical stability of the implant. It also contains cells in different amounts and types (i.e. neutrophils, lymphocytes, monocytes, and synovial lining cells) depending on the clinical state of the patient.^17^ However, the exact chemical and biochemical composition is much more complex and varies among different individuals including age,^18^ diet,^19^ and clinical state.^20^ All these compositional variations may produce a change in the chemical reactivity of the synovial fluid towards implanted biomaterials. Recent in vivo studies have highlighted variability in corrosion rate among patients, suggesting that individual patient factors might influence the synovial environment and, consequently, the reactivity of the implant material.^15,21^ Despite these findings, to our knowledge, no studies have investigated the relationship between clinical characteristics of patients and the electrochemical reactivity of their synovial fluid. Understanding these relationships could provide valuable insights into the mechanisms of implant corrosion and help to identify potential factors influencing implant reactivity and corrosion risks.

In this study, we aimed to explore the relationships between electrochemical properties of synovial fluid collected from knee osteoarthritic (OA) patients undergoing TKA and clinical and demographic data, such as age, BMI, sex, disease severity scores, quality of life, and patient-reported outcomes. We sought to identify potential factors influencing implant reactivity and corrosion risks.

## Methods

Knee OA patients were enrolled prospectively and consecutively in this study approved by the local ethics committee (Commission canonale d’éthique de la recherche sur l’être humain (CER-VD); 202002568) if they had to undergo TKA due to severe knee OA, after providing written informed consent.

**Fig. 1:**
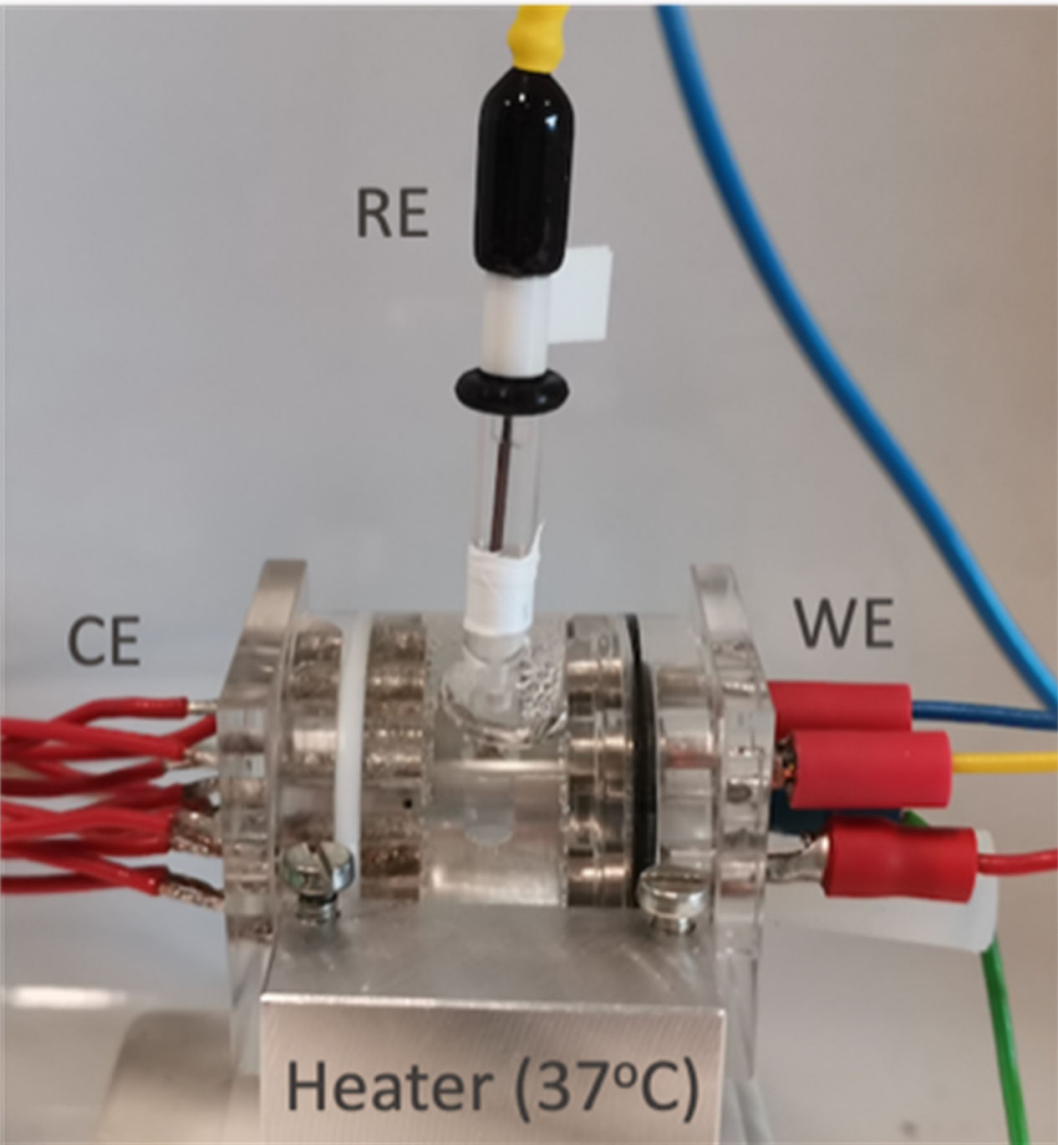
The three-electrode electrochemical cell used to carry out the in vivo electrochemical tests. CE, counter-electrode; RE, reference electrode; WE, working electrode.

During the TKA procedure, a sample of at least 2 ml of synovial fluid was collected from the patient’s joint by the same senior surgeon (BMJ). The sample was then directly transported to an adjacent room where it was immediately injected into a three-electrode electrochemical cell. The electrochemical cell was specifically designed to carry out electrochemical tests in vivo: for volumes of synovial fluid of 2 ml, sterilizable, oxygen-tight, and with temperature control (Figure 1). It allowed for testing four working electrodes in parallel (two titanium and two CoCrMo samples). The synovial fluid was directly injected into the cell from the upper part once received from the operating theatre. An Ag/AgCl electrode in a three molar potassium chloride saturated solution was used as reference electrode and ten Platinum wires as counter-electrode. A benchtop potentiostat (Ivium, Netherlands) was used to carry out a series of electrochemical tests. These tests consisted of measuring the open circuit potential (OCP, in volts) for 20 minutes, the polarization resistance (Rp) at the OCP, and the cathodic and anodic polarization curves. The OCP value was determined as the potential measured after 20 minutes of immersion, and it corresponds to the electrical potential difference between the Ti alloy and the synovial fluid measured with regard to the reference electrode. The corrosion rate of the Ti alloy was assessed using the polarization resistance method,^22^ performed every ten minutes by recording the current density while applying a small potential perturbation in both the anodic and cathodic directions and allowing for obtaining the corrosion rate of the titanium in each patient synovia. The polarization resistance (Rp in Ω·cm^2^) was then determined. Cathodic potentiodynamic curves from the OCP towards a cathodic potential of -1 V_AgAgCl_ at 2 mV/s were carried out in order to obtain the cathodic current density (i_c_ in mA/cm^2^) at -0.9 V_Ag/AgCl_. The details of the experimental protocol and set-up can be found in previous publications.^15,21^

A clinical evaluation of the patients was collected preoperatively to assess the severity of knee OA, the inflammation in the knee joint, the patient’s quality of life, and patient-reported outcomes. Specifically, the disease severity was evaluated using radiological Ahlbäck classification,^23^ based on weightbearing Rosenberg views. Visual inflammatory level was assessed by a trained clinician (BMJ) through a scale ranging from 0 to 4, 0 being without inflammation and 4 being the highest level of inflammation. The grading criteria for the different levels of visual inflammation are detailed in Table I. Quality of life was evaluated through the EuroQol measure for OA of the knee (EuroQol five-dimension questionnaire (EQ-5D)).^24^ Patient-reported outcomes were evaluated through the Western Ontario and McMaster Universities Osteoarthritis Index (WOMAC)^25^ and Knee Society Score (KSS).^26^ In addition, knee pain and stiffness were assessed using two separate visual analogue scales (VAS), each ranging from 0 (indicating no pain/stiffness) to 10 (indicating the worst imaginable pain/stiffness). Finally, a review of the patients’ medical histories was conducted. These histories were categorized based on their type: oxygen-reducing conditions (sleep apnoea syndrome, respiratory failure, asthma, osteonecrosis, thalassaemia, iron deficiency), structural injuries (fractures, ligament, or meniscal injuries), dyslipidaemia (diabetes, obesity), and osteoporosis. Patients were considered obese when their BMI was over 30 kg/m^2^.

**Table I.** Visual inflammation grading criteria.

Grade	Description
0	Normal-appearing synovial membrane with intra-articular fluid volume < 10 ml.
1	Normal-appearing synovial membrane with intra-articular fluid volume > 10 ml.
2	Hypertrophic inflammed synovial membrane with purplish appearance.
3	Hypertrophic, purplish, inflammed synovial membrane with intra-articular fluid volume > 10 ml.
4	Severely inflamed, dark purplish, and markedly hypertrophic synovial membrane.

A total of 43 patients were recruited for this study. There were 25 females and 18 males, with a mean age and BMI of 69.9 years (SD 7.6) and 27.6 kg/m^2^ (SD 5.2), respectively. They had a mean inflammation level and Ahlbäck grade of 3.0 (SD 0.8) and 3.2 (SD 0.5), respectively. Demographics and health status are detailed in Table II.

**Table II.** Demographics and health status for the osteoarthritic patients.

Variable	Data
Sex (F/M), n	25/18
Mean age, yrs (SD)	69.9 (7.6)
Mean BMI, kg/m^2^ (SD)	27.6 (5.2)
Mean Ahlbäck grade, range 1 to 5 (SD)	3.23 (0.53)
Mean inflammation, range 1 to 4 (SD)	3.02 (0.80)
Mean EQ-5D for osteoarthritis of the knee, range 0 to 1 (SD)	0.60 (0.27)
**Mean VAS, range 0 to 10 (SD)**	
Pain	5.7 (2.3)
Stiffness	4.4 (2.3)
**Mean WOMAC (SD)**	
Pain, range 0 to 20	9.5 (3.1)
Stiffness, range 0 to 8	3.7 (1.1)
Function, range 0 to 68	26.1 (10.0)
Total, range 0 to 96	39.3 (13.0)
**Mean KSS (SD)**	
Knee score, range 0 to 40	31.9 (5.4)
Knee function score, range 0 to 60	40.1 (13.3)
Total, range 0 to 100	72.0 (15.6)

EQ-5D, EuroQol five-dimension questionnaire; KSS, Knee Society Score; VAS, visual analogue scale; WOMAC, Western Ontario and McMaster Universities Osteoarthritis Index.

### Statistical analysis

To study the relationship between the electrochemical parameters and the demographic data, different tests were conducted. Specifically, Mann-Whitney U tests were performed to identify differences between males and females regarding the electrochemical parameters, and Spearman correlations were conducted to examine the relationship of age, BMI, inflammatory levels, and disease severity with the electrochemical parameters. To characterize the relationship between the electrochemical parameters and the clinical scores, including EQ-5D, VAS, WOMAC, and KSS scores, Spearman correlations controlled for age and BMI were conducted. Finally, to analyze whether there are differences in electrochemical parameters based on the category of medical history, subgroups of patients were created according to their medical history categories. Specifically, for each category of medical history, patients were divided into two groups: patients with a history of that category, and the remaining patients without a history of that category. If fewer than five patients had medical history of a particular category, that category was excluded from the analysis. For the remaining medical history categories, Mann-Whitney U tests were performed to compare the electrochemical parameters between patients with and without the medical history. The normality of the data was assessed using the Shapiro-Wilk test. Several variables, such as the electrochemical parameters, were found to be non-normally distributed, and thus non-parametric analyses were performed. Statistical significance level was set a priori at 5%. Statistics were done with Matlab version 2021a (MathWorks, USA).

## Results

The electrochemical procedures allowed us to get OCP values for all 43 patients, while Rp and i_c_ values could be extracted for 30 of the patients. Median values of the OCP, Rp, and i_c_ were -0.376 V (IQR 0.12), 1.39 × 10^6^ Ω·cm^2^ (IQR 1.48 × 10^6^), and -1.00 mA/cm^2^ (IQR 8.57), respectively. Mann-Whitney U tests showed no significant difference between male and female patients in any of the electrochemical parameters (p > 0.12). Spearman correlations showed no significant association between the electrochemical parameters and the age and BMI of the patients. Similarly, no association was found with the inflammation level. A significant correlation was found between Rp values and Ahlbäck scores (rho = 0.37, p = 0.044, Spearman correlations), indicating that the higher the disease severity grade, the larger the polarization resistance. Similarly, smaller i_c_ values were associated with higher Ahlbäck grades (rho = 0.41, p = 0.024, Spearman correlations). No correlation was found between the OCP and disease severity (Table III).

**Table III.** Spearman correlations between electrochemical data and demographics.

Variable	OCP		Rp		i_c_	
	Rho	p-value	Rho	p-value	Rho	p-value
Age	-0.07	0.634	0.05	0.780	-0.02	0.912
BMI	-0.29	0.056	-0.08	0.665	0.07	0.715
Inflammation	-0.21	0.178	0.12	0.512	0.15	0.418
Ahlbäck	-0.16	0.294	0.37	0.044	0.41	0.024

i_c_, cathodic current density; OCP, open circuit potential; Rp, polarization resistance.

Concerning the clinical scores, a high correlation was found between the Rp values and WOMAC stiffness scores (rho = 0.72, p = 0.002, Spearman correlations), indicating that the higher the WOMAC stiffness score, the larger the polarization resistance. A smaller KSS knee score was also associated with a lower OCP value (rho = 0.36, p = 0.025) and a less negative i_c_ value (rho = -0.39, p = 0.042, Spearman correlations). No significant association was found between other electrochemical parameters and clinical scores. All results are presented in Table IV.

**Table IV.** Spearman correlations between electrochemical data and clinical scores, controlled for age and BMI.

Variable	OCP		Rp		i_c_	
	Rho	p-value	Rho	p-value	Rho	p-value
EQ-5D for osteoarthritis of the knee	0.10	0.643	-0.16	0.566	-0.08	0.776
**VAS**						
Pain	-0.12	0.609	0.41	0.129	0.40	0.139
Stiffness	-0.13	0.605	0.49	0.062	0.45	0.094
**WOMAC**						
Pain	0.08	0.715	0.46	0.084	0.37	0.180
Stiffness	-0.06	0.795	0.72	0.002	0.47	0.077
Function	0.10	0.652	0.16	0.575	0.01	0.981
Total	0.09	0.694	0.27	0.325	0.16	0.560
**KSS**						
Knee	0.36	0.025	-0.29	0.141	-0.39	0.042
Function	-0.20	0.216	0.04	0.833	0.06	0.778
Total	-0.02	0.901	-0.08	0.691	-0.12	0.564

EQ-5D, EuroQol five-dimension questionnaire; i_c_, cathodic current density; KSS, Knee Society Score; OCP, open circuit potential; Rp, polarization resistance; VAS, visual analogue scale; WOMAC, Western Ontario and McMaster Universities Osteoarthritis Index.

**Fig. 2:**
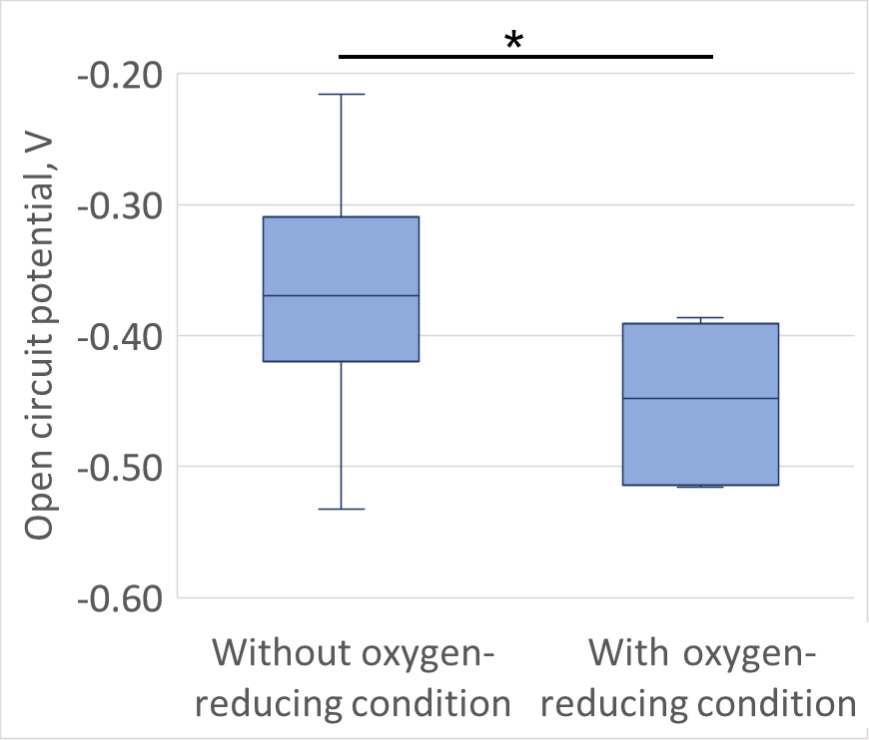
Boxplot of the open circuit potential values for patients with oxygen-reducing condition prior to surgery (right) and without such condition (left). The asterisk indicates a significant difference between the two groups of patients (p < 0.01, Mann-Whitney U test).

The search for clinical condition prior to the TKA conducted on the patient’s medical history found two categories of medical history with more than five patients: seven patients had oxygen-reducing conditions prior to surgery (five patients had respiratory failure, one had sleep apnoea syndrome, and one had minor thalassaemia), and 13 had dyslipidaemia (all had obesity). Group comparison showed that patients with oxygen-reducing conditions had significantly larger OCP values than patients without medical history in that category (p = 0.008, Mann-Whitney U test) (Figure 2). No difference was found for Rp (p = 0.979) and i_c_ (p = 0.938, Mann-Whitney U test) values. No difference was found between obese and non-obese patients for all three electrochemical parameters (all p ≥ 0.191, Mann-Whitney U test).

## Discussion

This study explored the relationship between the electrochemical properties of synovial fluid collected from patients undergoing TKA and their clinical and demographic characteristics. Our findings offer novel insights into the interplay between synovial fluid composition, patient-specific factors, and implant corrosion risks. For the first time, correlations between a patient’s clinical characteristics and an in vivo electrochemical measurement have been obtained.

The significant positive correlation between Rp and Ahlbäck scores suggests that patients with more advanced disease severity exhibit synovial fluid environments that reduce the electrochemical reactivity of titanium implants. This finding aligns with the hypothesis that changes in the biochemical composition of synovial fluid in advanced OA might modulate implant corrosion. Specifically, the higher Rp values observed in severe OA may indicate a protective effect against corrosion. Similarly, the observed relationship between i_c_ and Ahlbäck scores suggests a diminished cathodic reaction rate in patients with higher disease severity, highlighting once again the diminished reactivity of the synovial fluid in more severe patients. This finding highlights the complex electrochemical dynamics of synovial fluid, where biochemical changes linked to OA progression might influence specific corrosion pathways. Further biochemical analysis of the synovial fluid composition is needed to determine the underlying mechanisms driving these electrochemical trends.

The high correlation between Rp values and WOMAC stiffness scores emphasizes the potential link between patient-reported joint stiffness and synovial fluid’s electrochemical behaviour. This result goes in the same direction as the correlations with disease severity, with more symptomatic patients being at less risk of electrochemical reaction. Patients with higher stiffness scores may have synovial fluid compositions that increase Rp, offering some degree of protection against corrosion. These findings raise intriguing questions about the biochemical foundations of joint stiffness and their impact on implant material stability. It has been reported in literature that proteins decrease the kinetics of the oxygen reduction phenomena responsible for the corrosion by adsorbing on the metallic surfaces, thus leaving fewer sites for oxygen to react on.^27^ This phenomenon could explain why higher WOMAC stiffness scores have been associated with less corrosion reactivity. The correlations between the KSS knee score and OCP and i_c_ values reinforce these findings, as more symptoms were also associated with less reactivity (higher OCP values and lower i_c_ values). This may reflect an interaction between better joint functionality and a synovial environment that promotes oxygen reduction. These findings warrant further investigation into how clinical improvements in joint symptoms might influence implant corrosion risk. It is also important to note that no correlation was found with the other clinical scores, therefore it could be interesting to investigate further to establish why certain symptoms influence the reactivity, but not others.

The analysis of the patient’s medical history led to results which are worth discussing. The statistically significant larger OCP values among patients with oxygen-reducing conditions highlights the potential role of systemic conditions in modifying the synovial fluid’s electrochemical properties. Oxygen-reducing conditions, such as respiratory failure or sleep apnoea syndrome, may lead to hypoxic environments, which could affect the redox potential and subsequent implant reactivity. Previous results have shown that the amount of oxygen in the synovial fluid is a critical factor for corrosion of metallic implants.^28^ The difference of OCP found in the present study between patients with and without oxygen-reducing conditions corroborates the critical role of oxygen in corrosion. We can extend this interpretation with the correlations found with disease severity and symptoms, as the oxygenation of the synovial fluid of a more affected knee is decreased, potentially making it less corrosive to metallic implants.

The clinical relevance of this study lies in its potential to better identify patients at risk of implant corrosion based on their preoperative clinical and radiological profile. By establishing associations between synovial fluid electrochemical properties and patient-reported symptoms or systemic oxygenation status, the findings of this study offer valuable insights into how individual patient characteristics influence implant corrosion risk. Specifically, patients with fewer symptoms or better joint function may paradoxically present with a synovial environment more prone to corrosion, as reflected by lower Rp values or higher cathodic activity. If validated in larger and longitudinal cohorts, this information could contribute to preoperative risk stratification, personalized implant selection such as alternative materials for high-risk patients, or more targeted postoperative surveillance in patients likely to experience accelerated corrosion-related degradation. This study therefore opens the door to a more individualized approach to managing corrosion risk in TKA.

Our findings align with prior in vitro studies demonstrating that protein-rich biological environments can reduce corrosion by adsorbing on metallic surfaces, thereby limiting oxygen reduction but also altering other electrochemical reactions. For example, Talha et al^29^ reviewed the important influence of protein adsorption on the corrosion behaviour of metallic implants, highlighting that proteins can form protective films on implant surfaces, thereby inhibiting corrosion processes. These mechanisms may explain the higher Rp and OCP values, along with lower i_c_ values, observed in patients with more advanced OA or greater joint stiffness, conditions likely associated with altered synovial fluid composition. Furthermore, our findings of reduced synovial fluid corrosivity in patients with oxygen-reducing conditions introduce a novel perspective on the potential role of oxygen availability in modulating implant corrosion. To date, very few studies have reported in vivo electrochemical measurements of synovial fluid in OA patients. While this limits direct comparison with previous literature, it also highlights the novelty and potential value of the present study. Notably, the OCP, Rp, and i_c_ values measured in this study are within the expected range for metallic implants in biological fluids, and higher Rp and OCP values, as well as lower i_c_ values, are generally associated with more stable, less reactive surfaces, and therefore a reduced risk of corrosion.

This study has several limitations. The sample size was relatively modest, which may limit the ability to detect more subtle associations; nonetheless, several meaningful findings were identified, improving the understanding of how patients’ characteristics and disease severity influence synovial fluid corrosivity. Additionally, the study design was cross-sectional, focusing exclusively on preoperative clinical status and synovial fluid properties measured at the time of surgery. Further studies will be necessary to assess longitudinal changes or to determine whether synovial electrochemical parameters can predict postoperative outcomes or implant survival rates. Another potential limitation lies in the subjectivity inherent to several clinical assessments used in this study, including the visual grading of inflammation and patient-reported outcome measures. Although efforts were made to ensure consistency, such as relying on a single experienced observer and using validated personal reported outcomes, some degree of intraobserver variation and patient interpretation bias cannot be excluded. Finally, although correlations were observed with several clinical scores and oxygenation status, the underlying mechanisms driving these associations remain unclear. Future research incorporating proteomic or metabolomic analyses of the synovial fluid could help clarify the specific molecular mechanisms driving the observed electrochemical patterns. Despite these limitations, this study provides a novel perspective on the relationship between patient-specific factors and synovial fluid reactivity, and supports the need for future studies in this area.

In conclusion, this study highlighted that specific preoperative patient characteristics, including disease severity, joint stiffness, and the presence of oxygen-reducing conditions, are associated with the electrochemical reactivity of synovial fluid collected during TKA. Patients with more advanced radiological OA and more pronounced symptoms exhibited synovial environments with reduced corrosivity. Conversely, patients with fewer symptoms or better functional status presented with more electrochemically reactive synovial fluid, potentially increasing the risk of implant corrosion and metal ions release. These findings offer a novel perspective on how synovial fluid reactivity reflects clinical and radiological factors and may influence implant stability. Clinically, this introduces the possibility of identifying patients at higher risk for corrosion-related complications before implementation, paving the way for more personalized implant selection and postoperative monitoring. Further longitudinal and biochemical investigations are needed to validate these associations and explore their predictive value for long-term implant outcomes.

## Data Availability

The datasets generated and analyzed in the current study are not publicly available due to data protection regulations. Access to data is limited to the researchers who have obtained permission for data processing. Further inquiries can be made to the corresponding author.
